# ORGANIZATIONAL CULTURE AND SOCIAL ISOLATION IN NURSING HOMES

**DOI:** 10.1093/geroni/igae098.3876

**Published:** 2024-12-31

**Authors:** Ebenezer Martey, Jennifer Morgan, Antonius Skipper, Candace Kemp

**Affiliations:** University of Alabama at Birmingham, Birmingham, Alabama, United States; Georgia State University, Atlanta, Georgia, United States; Georgia State University, Atlanta, Georgia, United States; Georgia State University, Atlanta, Georgia, United States

## Abstract

Social isolation is a prevalent issue among nursing home residents, which can lead to various chronic diseases, such as depression, dementia, and chronic pain. Social integration refers to the active engagement in social ties, institutional connections, and participation in community-based activities. Organizational culture is a critical factor in providing person-centered care and fostering communication, teamwork, and quality improvement processes. Using a case study methodology, this study surveyed 49 nursing home staff and interviewed eight residents in one nursing center to understand the context of organizational culture and social integration among nursing home residents. Analysis of resident interview data indicated that communication challenges between staff and residents exist, as some residents report being uninformed, while others report great appreciation for staff responsiveness and personalized care. Social isolation was found to be a challenge for some residents due to a lack of meaningful engagement often exacerbated by health or mobility issues. Relationships between roommates often challenged resident well-being while supportive relationships among residents were essential to well-being. Residents noted that staff were very involved with encouraging residents to participate in activities and even take on specific responsibilities to foster a sense of community. The new availability of private rooms and bathrooms in this case study home was a big source of satisfaction for both residents and families. Private rooms appear to enhance satisfaction by enabling residents to exercise autonomy around daily schedules and promote dignity. Resident feedback on communication challenges revealed the need for organizations to practice greater transparency and consistent messaging.

